# The Paradox of Relief: Recognizing and Managing Re-expansion Pulmonary Edema Following Traumatic Hemothorax

**DOI:** 10.7759/cureus.95061

**Published:** 2025-10-21

**Authors:** Sri Hari Babu Sunkari, Ajay A, Vivek Kumar, Mahendra Chauhan

**Affiliations:** 1 Department of Trauma and Emergency, All India Institute of Medical Sciences, Nagpur, Nagpur, IND

**Keywords:** blunt thoracic trauma, chest tube, post-traumatic hemothorax, reexpansion pulmonary edema, white-out lung

## Abstract

A 20-year-old male presented two days after a road traffic injury with chest pain and breathlessness. Evaluation revealed a left-sided hemothorax with multiple rib fractures. Tube thoracostomy was done using a standard blunt dissection technique, under aseptic precautions, and drained 1100 mL of blood. Within two hours, the patient developed acute respiratory distress and hypoxia. Clinical and radiological findings, including diffuse crepitations, white-out of the left lung on chest X-ray, and consolidation with air bronchograms on CT, were consistent with re-expansion pulmonary edema (REPE). He was managed with noninvasive ventilation and supportive care in the intensive care unit. The patient improved steadily and was discharged on day 8. REPE is a rare complication of tube thoracostomy, particularly in trauma. This case highlights the importance of recognizing REPE early, even in acute settings, and emphasizes the need for cautious drainage of large-volume collections.

## Introduction

Re-expansion pulmonary edema (REPE) is an uncommon but potentially life-threatening complication that can occur after rapid re-expansion of a collapsed lung, typically following drainage of pneumothorax or pleural effusion. It is most frequently associated with chronic lung collapse lasting more than 72 hours. In the context of trauma, especially with hemothorax managed within a short time frame, REPE is exceedingly rare and may be overlooked. Despite its low incidence, estimated at around 0.01% [1], the clinical consequences can be severe if not promptly recognized. The exact pathophysiology of REPE remains incompletely understood, but it is believed to result from a combination of mechanical stress, ischemia-reperfusion injury, and inflammatory mediator release that collectively increase pulmonary capillary permeability [1,2], leading to non-cardiogenic pulmonary edema. The key message of this case is that REPE should remain on the differential diagnosis list in trauma patients who deteriorate following tube thoracostomy. Increased awareness can lead to timely intervention and improved patient outcomes.

## Case presentation

**Figure 1:**
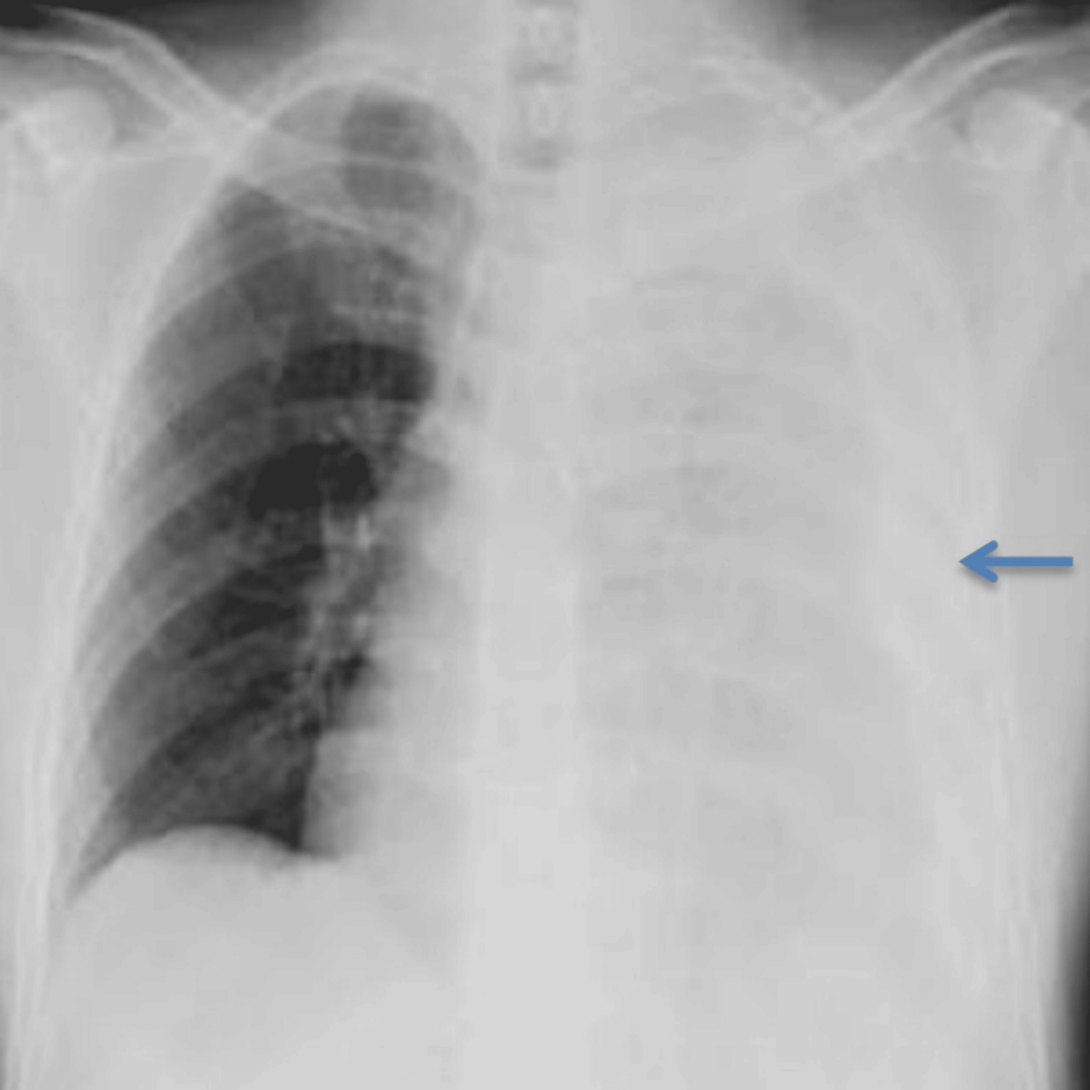
Initial chest X-ray (AP) at presentation showing a left-sided hemothorax with multiple rib fractures (arrow)

A 20-year-old male presented to the emergency department two days after sustaining blunt chest trauma during a road traffic incident. He reported left-sided chest pain, breathlessness, and right shoulder discomfort. On arrival, he was alert and oriented but had tachypnea. There was no significant past medical history, no prior surgery, and no relevant family history. He was a non-smoker with no allergies or history of chronic respiratory illness. Airway examination showed a patent airway. His respiratory rate was 26 breaths per minute, and oxygen saturation was 96% on 5 liters per minute of oxygen via face mask. Air entry was reduced on the left side of the chest, with dullness to percussion. His heart rate was 142 beats per minute, and his blood pressure was 106/88 mmHg. There was no evidence of external bleeding, pelvic injury, or long bone fractures. The Glasgow Coma Scale score was 15, and hypothermia was prevented during resuscitation. Focused chest examination revealed absent breath sounds over the left hemithorax. Heart sounds were normal. A chest radiograph demonstrated a left-sided hemothorax with multiple rib fractures (Figure 1). A diagnosis of traumatic hemothorax was made, and an intercostal chest drain was inserted using standard blunt dissection technique through the fifth intercostal space in the mid-axillary line under aseptic precautions, consistent with Advanced Trauma Life Support (ATLS) recommendations. Approximately 1,100 mL of blood was evacuated. Two hours later, the patient developed acute breathlessness, with oxygen saturation falling despite high-flow oxygen via a non-rebreather mask. Chest expansion was equal bilaterally, but diffuse crepitations were noted over the left lung field.

Investigations

Initial chest X-ray confirmed a left-sided hemothorax with multiple rib fractures (Figure 1), which guided the decision for tube thoracostomy. After chest drain insertion, arterial blood gas analysis showed type 1 respiratory failure with hypoxemia in the absence of hypercapnia.

**Figure 2:**
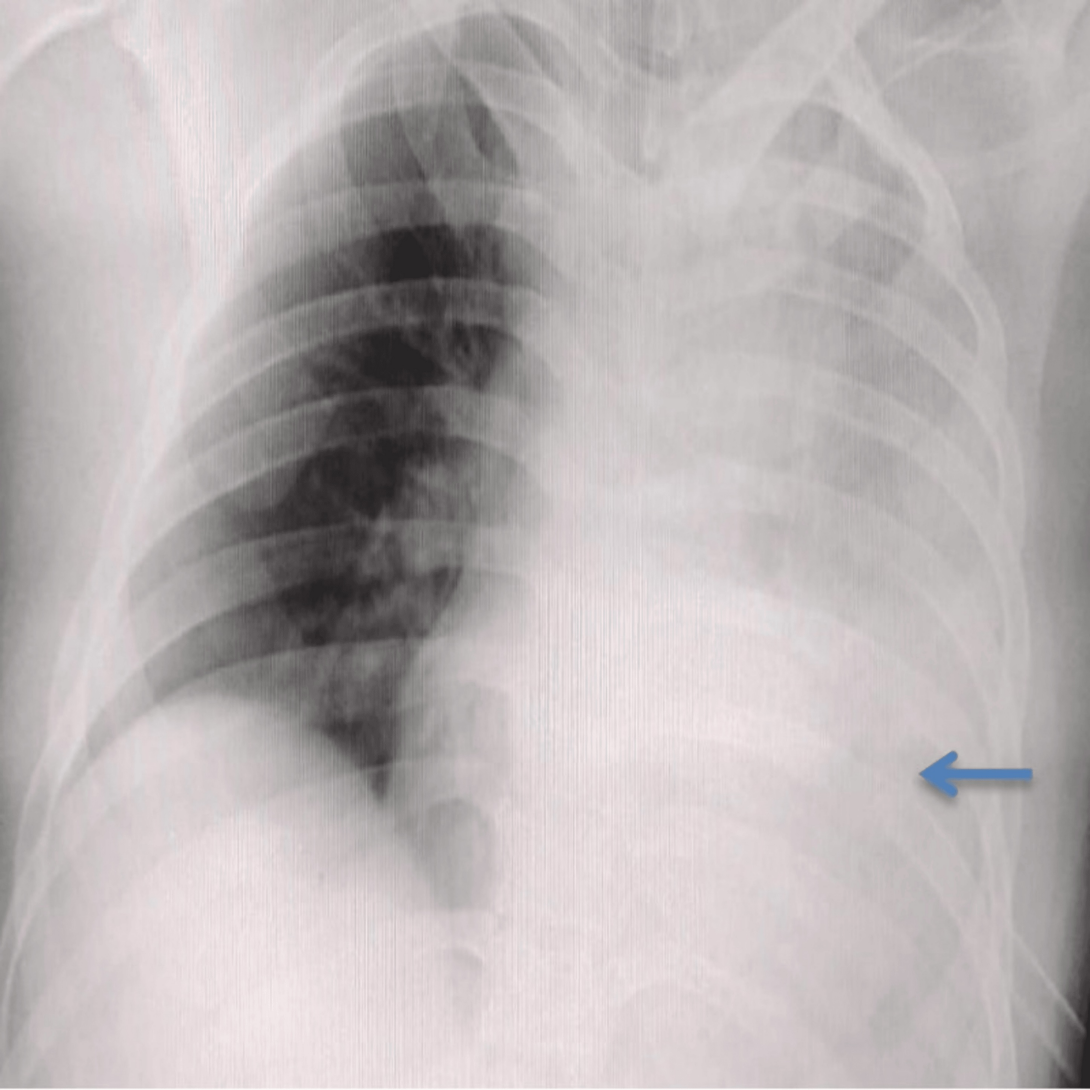
Chest X-ray (AP) obtained following clinical deterioration showing complete opacification (white-out lung) of the left hemithorax with the intercostal drainage tube in situ (arrow)

Following deterioration, a repeat chest X-ray revealed complete opacification of the left hemithorax (white-out lung) (Figure 2). Point-of-care ultrasound of the left lung field demonstrated confluent B-lines, suggestive of interstitial syndrome.

**Figure 3:**
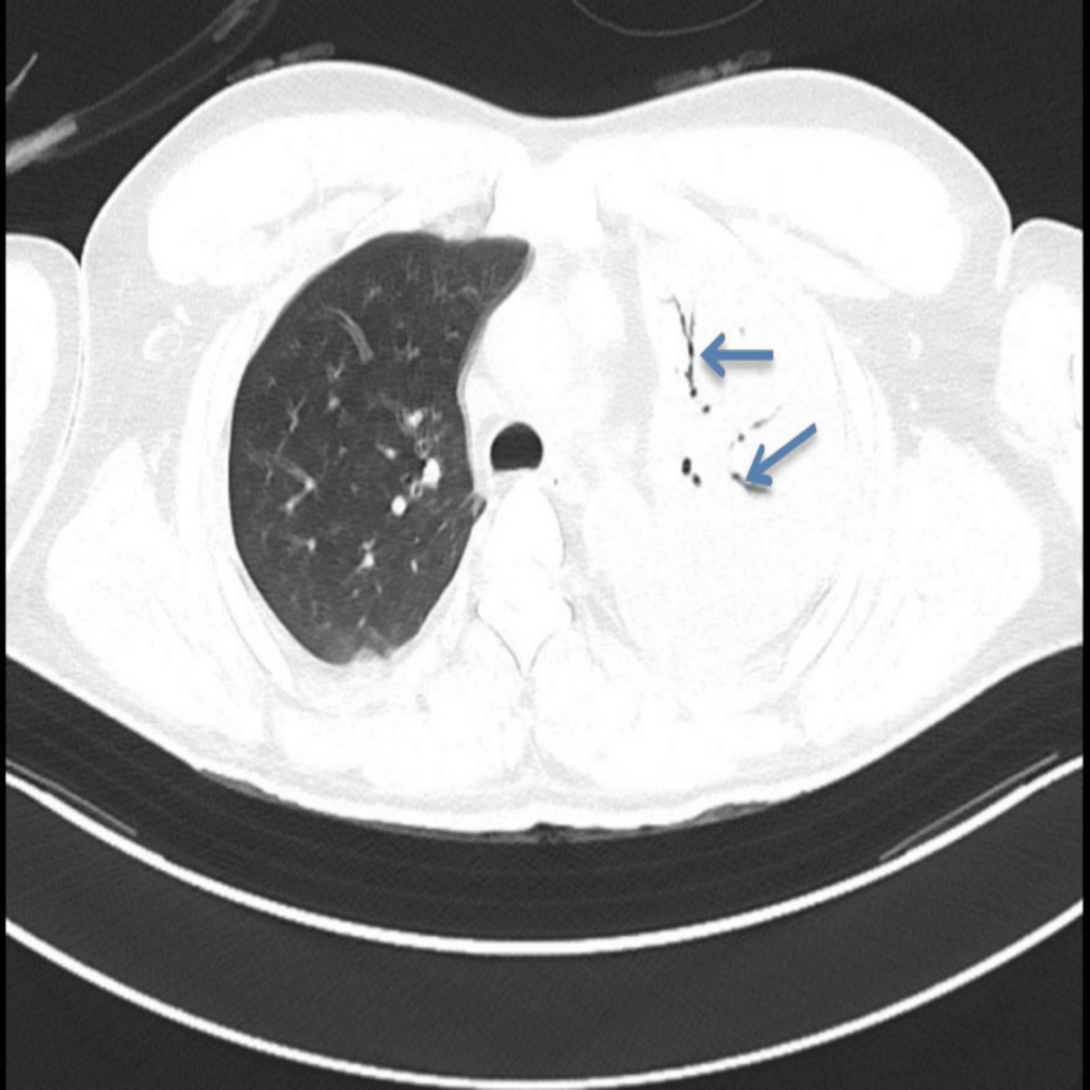
Contrast-enhanced CT thorax image (axial view) showing dense consolidation in the left lung parenchyma with air bronchogram (arrows)

A contrast-enhanced CT thorax further showed left lung consolidation with air bronchograms and excluded pneumothorax, re-accumulated hemothorax, pulmonary contusion, diaphragmatic injury, or endobronchial obstruction (Figure 3). These findings, combined with the acute clinical course and large-volume drainage, strongly supported the diagnosis of REPE.

Differential diagnosis

Several conditions were considered when the patient developed sudden respiratory distress following tube thoracostomy. Worsening hemothorax was ruled out, as there was no evidence of re-bleeding or increased pleural collection on repeat imaging, and drain output was minimal. Total atelectasis was unlikely because there was no mediastinal shift, and air bronchograms were preserved. Pneumonectomy was excluded, as there was no surgical history.

CT did not show typical features of lung contusion such as patchy ground-glass opacities. Endobronchial obstruction was excluded, as CT revealed no mass or mucous plugging. Iatrogenic lung injury from tube insertion was unlikely, as there was no continuous bubbling in the drain, no air leak, and no new collections. Diaphragmatic rupture and herniation were excluded by CT, which showed an intact diaphragm without herniated abdominal contents.

**Figure 4:**
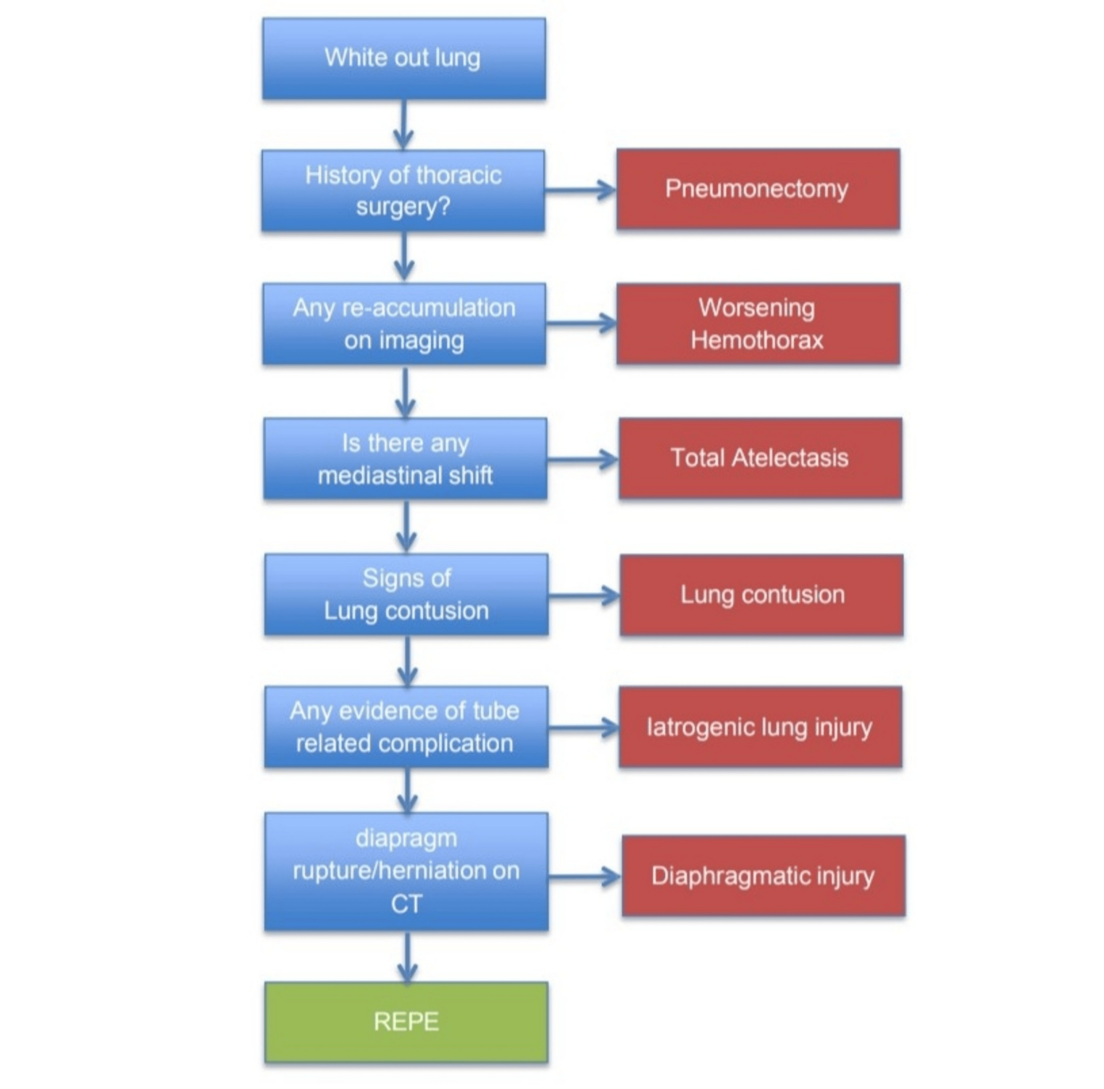
Flow diagram summarizing the differential diagnosis considered in this patient This figure highlights the systematic evaluation of possible causes of white-out lung, including atelectasis, iatrogenic hemothorax/lung injury, lung contusion, diaphragmatic injury, and REPE. Such an approach is crucial to differentiate REPE from other causes, ensuring timely recognition and appropriate management [1,3].

After excluding these conditions, the temporal association with drainage, acute onset of respiratory distress, radiological white-out lung, and consolidation with air bronchograms was strongly suggestive of REPE (Figure 4).

Treatment

The patient was immediately transferred to the intensive care unit for close monitoring and non-invasive ventilatory support. He was placed on bilevel positive airway pressure (BiPAP) with an inspiratory pressure of 12 cm H₂O and expiratory pressure of 6 cm H₂O. Oxygen was titrated to maintain saturation above 94%, guided by arterial blood gas monitoring. Strict fluid balance was maintained, and the patient was kept in a head-elevated position to optimize respiratory mechanics.

Analgesia was provided with intravenous paracetamol, and gastrointestinal prophylaxis was given with intravenous pantoprazole. No diuretics or corticosteroids were administered, as evidence does not support their routine use in REPE. Antibiotics were not prescribed, as there was no suspicion of infection. Physiotherapy and incentive spirometry were initiated once the patient’s condition stabilized. No further surgical intervention was necessary.

Outcome and follow-up

**Figure 5:**
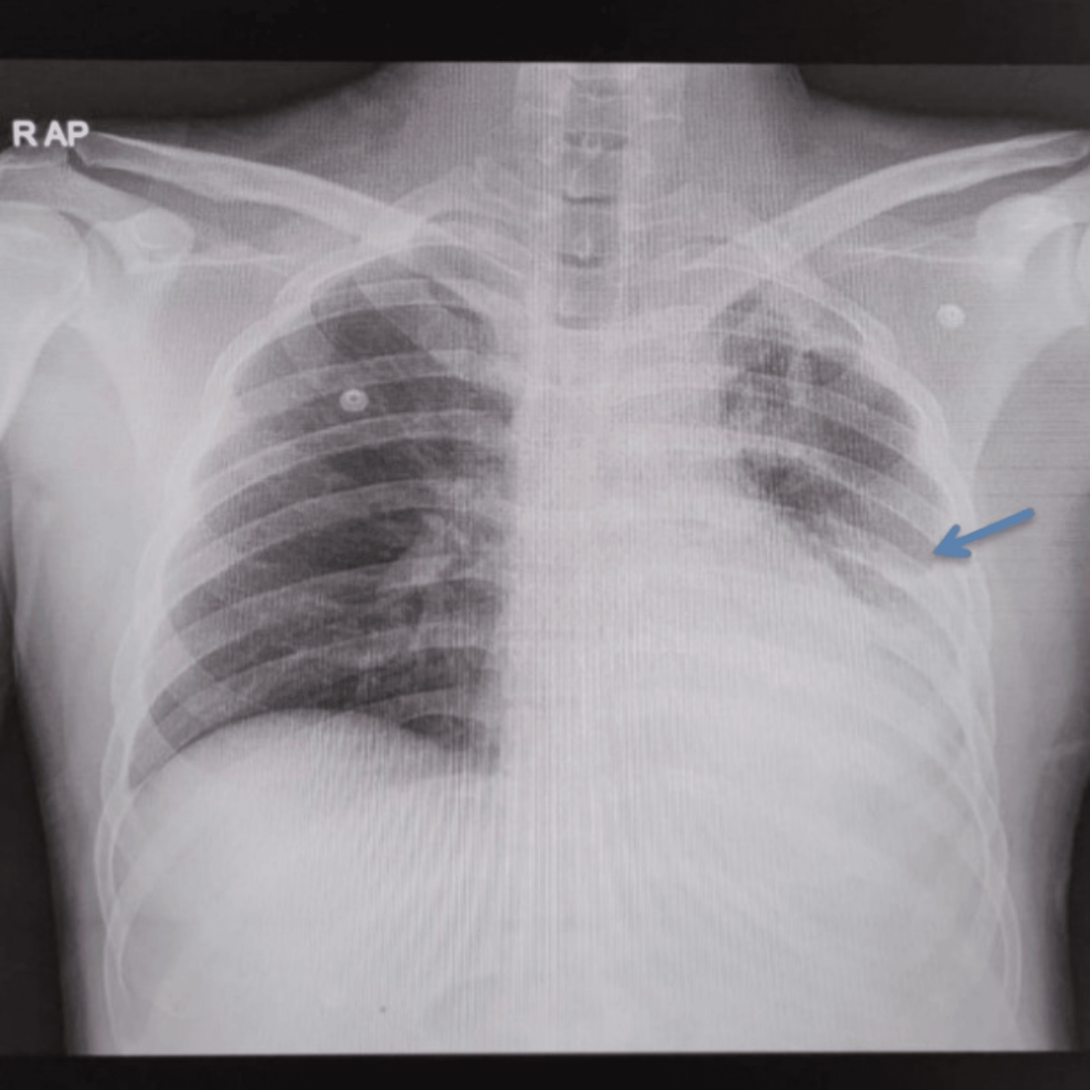
Chest X-ray (AP) revealing significant resolution of REPE and near-complete re-expansion of the left lung (arrow) AP: anteroposterior

Over the next 48 hours, the patient showed steady improvement in oxygenation and work of breathing. By day three, crepitations had decreased, and he tolerated intervals off non-invasive ventilation. By day five, he was maintaining oxygen saturation on room air, and non-invasive ventilation was discontinued. The intercostal drain was non-productive and removed on day seven. A chest X-ray at this stage showed marked improvement (Figure 5).

He was discharged on day eight in stable condition, breathing spontaneously with normal oxygen saturation. To provide a clear overview of the patient's progress and the timing of the key interventions, a chronological summary of clinical events is shown in Table 1. 

**Table 1:** Chronological summary of clinical events and interventions CXR = Chest X-ray, CECT = Contrast-enhanced CT, ABG = Arterial blood gas, ICD = Intercostal chest drain, NIV = Non-invasive ventilation

Timeline	Clinical Events / Findings	Interventions
Day 0 (Injury)	Road traffic injury with blunt chest trauma. The patient developed left-sided chest pain and breathlessness.	No immediate hospital visit.
Day 2 (Emergency Department presentation)	Examination and initial investigations revealed a massive left hemothorax with multiple rib fractures.	Tube thoracostomy performed in the fifth intercostal space (mid-axillary line) using a blunt dissection technique; 1,100 mL of blood was evacuated.
Day 2 (2 hours post-procedure)	Sudden respiratory distress, tachypnea, and desaturation.	High-flow oxygen was provided via a non-rebreathing mask. Repeat CXR showed “white-out” of the left lung. Patient stabilized and shifted for CECT thorax.
Day 2 (Post deterioration)	CECT thorax showed left-sided consolidation with air bronchograms; no new collection. ABG consistent with type 1 respiratory failure.	Provisional diagnosis of REPE was made, and NIV support was started.
Days 3–7	Gradual clinical improvement; stable oxygenation.	Weaning from NIV and ICD removal once drainage subsided.
Day 8	Symptom-free; lung expansion satisfactory.	Discharged with follow-up advice.

At a two-week follow-up, he reported complete symptom resolution, and a chest X-ray showed significant radiological improvement. By six weeks, he had resumed normal physical activity, with near-complete radiological resolution and no functional limitation. He was counseled about warning symptoms and advised to avoid strenuous exertion temporarily.

## Discussion

REPE is a rare but potentially life-threatening complication that may occur following the rapid re-expansion of a chronically collapsed lung. While it is most commonly reported in cases involving spontaneous pneumothorax or chronic pleural effusions, REPE is infrequently associated with traumatic hemothorax. The current case underscores this uncommon presentation and emphasizes the importance of recognition in acute care settings.

The precise mechanism of REPE is not fully understood but is believed to be multifactorial. Proposed mechanisms include mechanical stress from rapid lung re-expansion, ischemia-reperfusion injury, and inflammatory cytokine release, such as TNF-α and interleukins, which increase capillary permeability [1,2]. These processes culminate in non-cardiogenic pulmonary edema, typically unilateral and localized to the previously collapsed lung, though bilateral involvement has also been reported [4,5].

Risk factors described in the literature include younger age, large-volume drainage greater than 1,000 mL, prolonged lung collapse lasting more than three days, and rapid evacuation of pleural contents [6,7]. Our patient presented 48 hours post-injury, with approximately 1,100 mL of blood evacuated rapidly. This combination of risk factors may have precipitated REPE in this trauma setting, even though the collapse was less than 72 hours in duration.

REPE is primarily a diagnosis of exclusion. In this case, the development of acute respiratory distress within two hours of intercostal drainage, along with chest imaging showing white-out of the left lung and air bronchograms, raised a strong clinical suspicion. Alternate causes of unilateral opacification, including worsening hemothorax, pulmonary contusion, atelectasis, and iatrogenic injury, were systematically ruled out through CECT thorax, point-of-care ultrasound, and assessment of intercostal drain output. Arterial blood gas analysis revealed hypoxemia consistent with type 1 respiratory failure. This diagnostic approach aligns with published pathways, which emphasize recognition of temporal correlation, identification of imaging patterns such as preserved lung volume and absence of mediastinal shift, and systematic exclusion of alternative causes [4,8].

There are no formal international guidelines dedicated to REPE; however, management strategies are well-documented in the clinical literature. Treatment is primarily supportive, with interventions guided by severity. Mild cases may recover with oxygen therapy alone, while moderate to severe cases, such as in this patient, may require non-invasive ventilation or, in rare instances, intubation [6,9,10]. Pharmacological therapies, such as diuretics or corticosteroids, are not routinely recommended due to limited evidence. In this case, supportive care with non-invasive ventilation, fluid monitoring, analgesia, and physiotherapy led to complete recovery without the need for pharmacologic agents specifically targeting pulmonary edema. Preventive strategies described in elective settings include controlled and gradual drainage of pleural contents and limiting initial evacuation to 1-1.5 liters, especially when effusions or pneumothorax are long-standing [7,10]. In trauma cases, however, immediate decompression may be unavoidable, as was appropriate in this patient, given his respiratory compromise on presentation.

Comparison with existing literature

Mahfood et al. first described the clinical spectrum of REPE, while Neustein and Sohara documented its rapid onset after thoracic drainage [1,2,4]. Chakraborty et al. and Jayalakshmi et al. reported trauma-related cases with prolonged collapse beyond 72 hours, all showing favorable outcomes with early recognition and supportive care [5,10]. Kasmani et al. emphasized that REPE can follow even therapeutic thoracentesis [11]. In contrast, the present case is distinctive because REPE occurred after evacuation of a hemothorax that had been present for less than 72 hours, demonstrating that rapid high-volume drainage can precipitate REPE even without chronic lung collapse. This finding expands the known risk spectrum and supports careful monitoring even in relatively acute presentations.

In contrast, the present case is distinctive because REPE occurred after evacuation of a hemothorax that had been present for less than 72 hours, demonstrating that rapid high-volume drainage can precipitate REPE even without chronic lung collapse. This finding expands the known risk spectrum and supports careful monitoring even in relatively acute presentations. A comparison of published case reports with the present case is summarized (Table 2).

**Table 2:** Comparison of different case reports with the present study

Author (Year)	Underlying condition	Volume drained (mL)	Time to onset	Management	Outcome	Comments
Mahfood et al. (1988) [1]	Chronic pneumothorax/effusion	1,000–2,000	1–3 h	Oxygen, supportive	Full recovery	Prolonged collapse >72 h
Neustein (2007) [2]	Post-operative pneumothorax	~1,300	~2 h	Intubation	Full recovery	Chronic collapse
Sohara (2008) [4]	Chronic pneumothorax	>1,000	Hours	Oxygen, NIV	Full recovery	Chronic collapse
Chakraborty et al. (2011) [5]	Traumatic hemothorax	>1,000	1–3 h	NIV, supportive	Full recovery	Collapse ~3 days
Matsuura et al. (1991) [6]	Mixed pneumothorax/effusion	800–2,000	0.5–6 h	Oxygen/ventilation	Full recovery (majority)	Many >72 h
Hasegawa et al. (1993) [7]	Chronic pneumothorax (2 cases)	1,000–1,500	1–2 h	Oxygen, supportive	Full recovery	Prolonged collapse
Verhagen et al. (2014)[9]	Spontaneous pneumothorax	1,200	2 h	Oxygen, supportive	Full recovery	Chronic collapse
Jayalakshmi et al. (2022) [10]	Chronic pneumothorax	~1,000	1–3 h	NIV, supportive	Full recovery	Prolonged collapse
Kasmani et al. (2010) [11]	Large pleural effusion	~1,500	<2 h	Oxygen, supportive	Full recovery	Chronic effusion
Present case (2025)	Traumatic hemothorax	1,100	2 h	NIV, supportive	Full recovery	Developed REPE despite drainage <72 h after collapse—uncommon finding

Guidance from trauma and thoracic care literature recommends follow-up imaging within four to eight weeks and monitoring for long-term sequelae such as restrictive lung changes or pleural fibrosis. In our case, chest X-rays at two and six weeks showed complete radiologic resolution. The patient remained symptom-free and had resumed full daily activities, including a return to work, with no functional limitations.

## Conclusions

REPE should be suspected in trauma patients who develop sudden respiratory distress after rapid drainage of a large-volume hemothorax. It is important to promptly differentiate REPE from other causes of unilateral white-out lung, such as lung contusion, persistent hemothorax, or iatrogenic injury, which often requires the use of CT imaging. Supportive management and close post-procedure monitoring remain critical, as early recognition of REPE can significantly improve outcomes.
